# A DArT platform for quantitative bulked segregant analysis

**DOI:** 10.1186/1471-2164-8-196

**Published:** 2007-06-28

**Authors:** Peter Wenzl, Harsh Raman, Junping Wang, Meixue Zhou, Eric Huttner, Andrzej Kilian

**Affiliations:** 1 Diversity Arrays Technology P/L, 1 Wilf Crane Cr., Yarralumla, Canberra ACT 2600, Australia; 2 Triticarte P/L, 1 Wilf Crane Cr., Yarralumla, Canberra ACT 2600, Australia; 3 NSW Agricultural Genomics Centre and NSW Department of Primary Industries, Wagga Wagga Agricultural Institute, PMB, Wagga Wagga NSW 2650, Australia; 4 Tasmanian Institute of Agricultural Research, University of Tasmania, PO Box 46, Kings Meadows TAS 7249, Australia

## Abstract

**Background:**

Bulked segregant analysis (BSA) identifies molecular markers associated with a phenotype by screening two DNA pools of phenotypically distinct plants for markers with skewed allele frequencies. In contrast to gel-based markers, hybridization-based markers such as SFP, DArT or SNP generate quantitative allele-frequency estimates. Only DArT, however, combines this advantage with low development and assay costs and the ability to be deployed for any plant species irrespective of its ploidy level. Here we investigate the suitability of DArT for BSA applications using a barley array as an example.

**Results:**

In a first test experiment, we compared two bulks of 40 Steptoe/Morex DH plants with contrasting pubescent leaves (mPub) alleles on chromosome 3H. At optimized levels of experimental replication and marker-selection threshold, the BSA scan identified 433 polymorphic markers. The relative hybridization contrast between bulks accurately reflected the between-bulk difference in the frequency of the mPub allele (r = 0.96). The 'platform noise' of DArT assays, estimated by comparing two identical aliquots of a DNA mixture, was significantly lower than the 'pooling noise' reflecting the binomial sampling variance of the bulking process. The allele-frequency difference on chromosome 3H increased in the vicinity of mPub and peaked at the marker with the smallest distance from mPub (4.6 cM). In a validation experiment with only 20 plants per bulk we identified an aluminum (Al) tolerance locus in a Dayton/Zhepi2 DH population on chromosome 4H with < 0.8 cM precision, the same Al-tolerance locus that had been mapped before in other barley populations.

**Conclusion:**

DArT-BSA identifies genetic loci that influence phenotypic characters in barley with at least 5 cM accuracy and should prove useful as a generic tool for high-throughput, quantitative BSA in plants irrespective of their ploidy level.

## Background

Bulked Segregation Analysis (BSA) is a widely used method for rapidly identifying molecular markers linked to a trait of interest. It involves genotyping two pools (bulks) of DNA samples from individual plants originating from an experimental cross. Plants are assigned to one of the two bulks based on the trait of interest. The bulks are screened with a large number of markers to identify those that distinguish the bulks and, by inference, must be genetically linked to the trait locus [1].

The success of the BSA approach relies on the dramatic reduction in the number of marker assays when compared to building a genetic map for the purpose of identifying markers associated with a phenotype. BSA 'collapses' the two-dimensional matrix of marker assays (DNA samples × markers) into a one-dimensional vector of genotypic differences between two DNA bulks. With gel-based marker technologies this vector is largely built up sequentially. Highly multiplexed, hybridization-based marker technologies such as SFP, DArT and SNP have the potential to further 'collapse' the vector of genotypic differences between bulks into a single (perhaps replicated) whole-genome assay [2-5].

Application of hybridization-based marker technologies to BSA not only reduces the genotyping effort, but has the additional benefit of producing quantitative raw data (hybridization intensities) which are only subsequently converted into discrete genotypes (allele calls) in the case of non-BSA applications. The raw hybridization data are a quantitative measure of allele frequencies [6-8] and thus should increase the accuracy of mapping a trait locus with BSA. Others have used this feature when performing BSA experiments using SFP typed on Affymetrix GeneChips [9,10].

While SFP are a powerful research tool for species with sufficient sequence information, their utility in the context of agricultural research and (pre)breeding appears to be fairly limited, not only because of technology-establishment and per-sample assay costs but also because polyploidy poses a serious barrier to any whole-genome hybridization approach. Polyploidy and the costs of technology establishment for new species are also limiting the widespread deployment of SNP among the approximately 150 crop species cultivated worldwide, many of which have large and/or polyploid genomes [11,12]. By contrast DArT arrays, already available for two dozens of plant and fungal species [4,13-18], can be rapidly developed for new species of any ploidy level for a small fraction of the investment required for SFP or SNP arrays [19].

In this paper we investigate the suitability of the DArT platform for BSA, using as an example a polymorphism-enriched barley array with 2,304 clones [20]. We first test 'DArT-BSA' using a DH population that segregates for a morphological marker with known map position (our model target trait). In this experiment we explore several technology features that are likely to be critical for routine deployment of DArT-BSA. In a subsequent validation experiment we deploy the optimized method to a different DH population segregating for Al tolerance to test the performance of DArT-BSA in a practical application.

## Results and discussion

### Test experiment

The Steptoe/Morex DH population segregates for 'pubescent leaf blades' (mPub), a morphological marker that has been incorporated into the genetic map of chromosome 3H in this population [21]. We used mPub to assemble contrasting bulks for a trait with known genetic location in order to evaluate the performance of DArT when applied to BSA. The bulks were compared by simultaneously assaying them on the same DArT array (replicated up to eight times) and measuring the contrast of hybridization intensity for markers that were identified as polymorphic in a comparison between the two parents on separate replicated arrays.

#### Selection of polymorphic markers

Markers differentiating between Steptoe and Morex were selected based on the contrast in hybridization intensity between the two cultivars (log_2 _[cy3/cy5]). Instead of applying an arbitrary threshold we measured the variability of hybridization-contrast estimates by comparing two identical aliquots of a 1:1 mixture of the two parents ('self comparison'). Polymorphic markers were then selected by applying to the hybridization contrast between the parents a normal distribution-based probability threshold derived from the self comparison.

As the stringency of this marker-selection threshold was increased from 10^-2 ^to 10^-5^, the minimum parental hybridization contrast increased from 0.69 to 1.18 log units (Table 1). The latter caused a concomitant decrease in the number of selected polymorphic markers from 631 to 384. At the same time the proportion of markers previously mapped in the Steptoe/Morex population with an array containing a subset of markers [4] increased from 45 to 55% (Table 1). These numbers indicate a gradual enrichment of high-quality, 'mappable' markers at more stringent thresholds at the expense of excluding other possibly informative markers. A test of several threshold levels for their effectiveness in excluding outlier markers in a plot of relative hybridization contrast vs. map position (see section entitled *Genome-wide linkage scan *for an example) suggested that *p *< 0.0001 was an acceptable compromise between these two tendencies (data not presented). This threshold was used for the rest of this study.

**Table 1:** Effect of experimental settings on polymorphic-marker selection and linkage-detection thresholds in the mPub BSA scan

						Linkage-detection threshold (p < 0.05)^5^	
						
Bulk size	Number of replicate arrays	Marker-selection p threshold^1^	Minimum hybridization difference between parents^2^	Number of polymorphic markers identified^3^	Markers previously mapped in Steptoe/Morex population^4^	Based on 'platform noise'^6^	Based on 'pooling noise'^7^
20	4	0.001	0.88	515	257	26%	50%
20	4	0.0001	1.04	433	231	24%	50%
20	4	0.00001	1.18	384	211	23%	50%
20	2	0.0001	1.06	356	187	24%	50%
20	8	0.0001	0.66	669	294	24%	50%
40	4	0.0001	1.04	418	221	23%	37.5%

^1^A normal distribution-based threshold for log2 [cy3/cy5] derived from the comparison of two identical aliquots of a 1:1 mixture of the Steptoe and Morex parents.

^2^log2 [cy3/cy5]

^3^Markers were selected from the set of 2,304 polymorphism-enriched clones (see section entitled 'DArT assays' in Materials and Methods).

^4^DArT markers were mapped on an array containing a partly overlapping set of markers.

**Figure 1:**
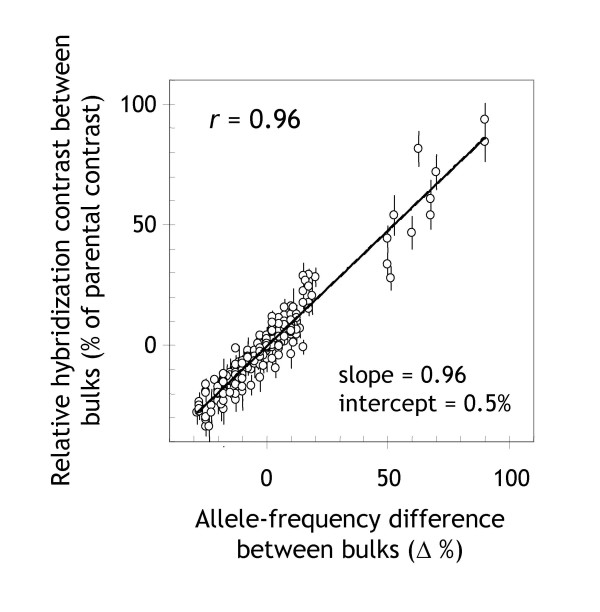
**Quantitative precision of DArT-BSA**. For each marker, there is a 1:1 relationship between the relative hybridization contrast (= log_2 _[cy3/cy5] between bulks as a percentage of log_2 _[cy3/cy5] between parents) and the allele-frequency difference between the bulks. This relationship makes interpretation of experimental results straightforward. The plot is based on a comparison of *mPub *bulks (size = 40), prepared from genomic representations of individual Steptoe/Morex DH plants (see corresponding genome scan in Figure 3). It includes all markers present in a previously published Steptoe/Morex DArT map [20] and reports the difference in the frequency of Steptoe alleles between bulks.

^5^Values are based on the dispersion of the relative hybridization contrast (log2 [cy3/cy5] as a percentage of log2 [cy3/cy5] measured in the parental comparison) or the allele-frequency difference. There was a 1:1 correspondence between the two (Figure 1).

^6^This significance threshold reflects the variability inherent in the array-hybridization process. It was derived from the dispersion of the relative hybridization contrast in a 'self' comparison between two identical aliquots of 1:1 mixture of Steptoe and Morex (= ratio between log2 [cy3/cy5] in the self and the parental comparison). The resulting significance threshold was Bonferroni-adjusted for multiple comparisons.

**Figure 2:**
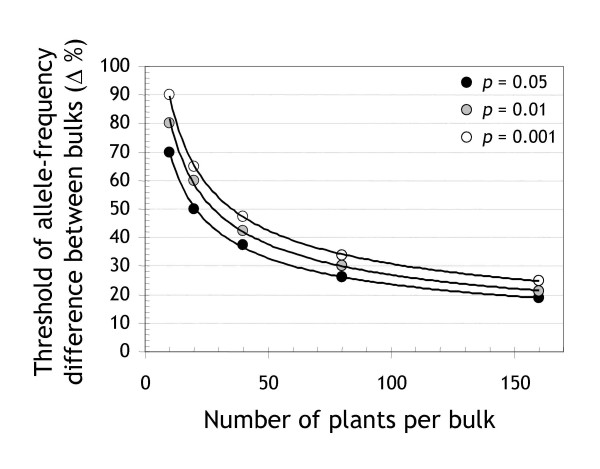
**'Pooling noise': the effect of bulk sizes on the amplitude of spurious linkage signals**. Genome-wide significance thresholds for detecting spurious linkage between a marker and a target locus were derived from 10,000 simulated comparisons between bulks of F_1_-derived DH barley plants (see section entitled 'Allele-frequency determination and simulation' in *Materials and Methods *for details). Trait-linked markers should only be considered as statistically significant if the allele-frequency difference between bulks is superior to the value derived for the relevant combination of bulk size and desired probability level.

^7 ^This significance threshold reflects the chance that a non-zero allele-frequency difference may occur by chance as a result of the random assortment of chromosomes (and unlinked areas within chromosomes) in the pooling process. It was derived by simulating the pooling process (see section entitled 'Allele-frequency determination and simulation' in *Materials and Methods *and Figure 2).

#### Experimental replicates

We next tested the effect of varying the number of replicate arrays between two and eight. Increasing the degree of replication resulted in the inclusion of markers that were previously not mapped, in part because the hybridization contrast between the parental alleles was small (Table 1) [20]. Only a small minority of the additional markers, however, were outliers with respect to a Loess curve in a plot of the relative hybridization contrast vs. map position (see section entitled *Genome-wide linkage scan *below for an example). This result suggests that most of these markers were still genetically informative. A high degree of experimental replication seems to improve the precision of quantifying markers with a smaller hybridization contrast between the parental alleles, thus resulting in the inclusion of more markers in a BSA scan. We considered four experimental replicates adequate for the purpose of this study.

#### Quantification of the allele-frequency difference between bulks

The contrast in hybridization intensity between the two allelic states varies from marker to marker and has to be taken into account when quantifying allele abundances in DNA pools. The log2 [cy3/cy5] values measured for polymorphic markers in the bulk comparison were therefore scaled by division by the log2 [cy3/cy5] values measured in the comparison between the parents.

To link the scaled or relative hybridization contrast of a marker to the allelic composition of the bulks, we computed from the segregation data of a Steptoe/Morex DArT map the frequency of the Steptoe allele in each bulk (see section entitled 'Allele-frequency determination and simulation' in Materials and Methods). The difference between the Steptoe allele frequencies in the two bulks was closely correlated to the relative hybridization contrast (r = 0.96; Figure 1). The degree of correlation was identical to the correlation obtained in other studies using the Affymetrix SNP genotyping platform [7,8]. This result confirms that hybridization intensities are proportional to the abundance of DArT alleles in DNA pools. The relative hybridization contrast, therefore, can be used as a measure of the between-bulk difference in the frequency of the allele from the trait-donor parent.

It follows from this data that previously identified limitations of dominant markers for BSA with certain population types [22] do not apply to DArT-BSA, although in a non-BSA context DArT markers are typically scored in a dominant manner [13]. Any type of population that segregates for a trait of interest should be amenable to DArT-BSA; be it DH, RIL, BC, F2 or more complex populations.

#### Linkage-detection threshold derived from 'platform noise'

Before scanning the genome for markers linked to *mPub *we quantified the basal 'platform noise' to obtain a significance threshold for detecting linkage. The dispersion of the apparent allele-frequency difference in a comparison between two identical aliquots of a 1:1 mixture of the parents was used to derive a genome-wide significance threshold (Table 1). The size of this threshold was a function of the extent to which markers with limited hybridization contrast between alternative alleles were incorporated in the analysis (Table 1), because the dispersion of allele-frequency estimates was larger for less well-separated markers (Additional File 1). More experimental replicates kept the threshold low even if poorly separated markers were included (Table 1).

#### Linkage-detection threshold derived from 'pooling noise'

Besides quantifying the effect of platform-related noise, it is important to consider the expected variability caused by binomial sampling in the bulking process. As a result of the random assortment of chromosomes, the between-bulk difference in allele frequency may deviate from zero for markers that are not linked to the target locus, thus generating spurious linkage signals.

We established a genome-wide significance threshold by simulating the comparison of random bulks (see *Materials and Methods *for details). With increasing bulk sizes, the threshold asymptotically drops toward 0% allele-frequency difference (Figure 2). At 40 plants per bulk, for example, there is a 5% probability of detecting, by chance only, at least one genomic region with an allele-frequency difference greater than 37.5% (Table 1). The thresholds reported in Figure 2 are specific to the number of chromosomes and the type of population used in this study. For species with more chromosomes, for example, larger bulk sizes are required to achieve comparable thresholds (data not presented).

#### Genome-wide linkage scan

**Figure 3:**
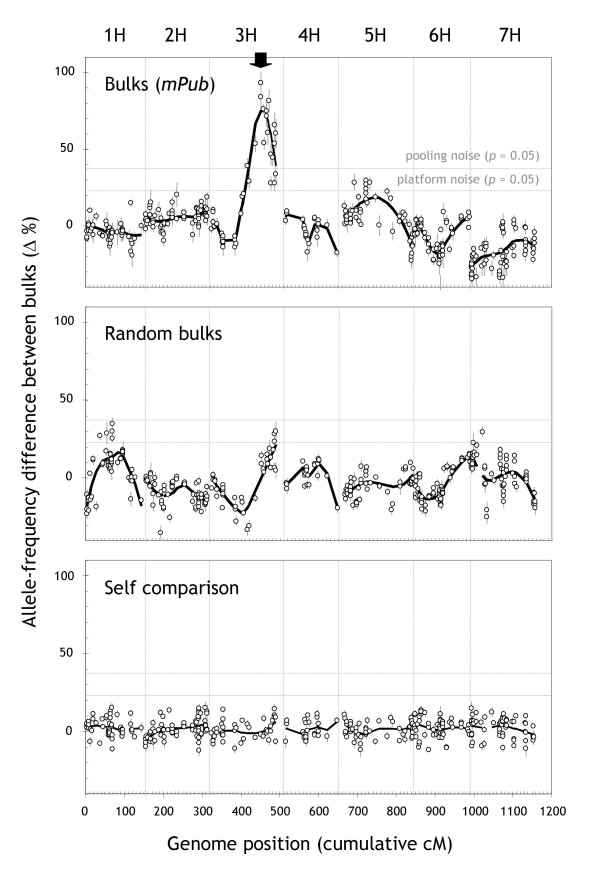
**DArT-BSA genome scan for the 'pubescent leaves' (*mPub*) locus in the Steptoe/Morex DH population**. The difference in the frequency of Steptoe alleles between different pairs of DNA pools is shown as a function of the cM positions of markers previously incorporated into a DArT consensus map for barley (375 of 418 polymorphic markers) [20]. The '**Bulks (*mPub*)**' panel displays data from the comparison between bulks contrasting for the *mPub *locus on chromosome 3H (40 plants each). The black arrow indicates the position of the *mPub *locus on the DArT consensus map [20] according to which the DH plants had been distributed into bulks. The '**Random bulks**' panel displays data from a comparison of two randomly assorted bulks of 40 plants each. The '**Self comparison' **panel shows the result of a comparison of two identical aliquots of a 1:1 mixture of Steptoe and Morex. Vertical lines within each of the panels denote borders between individual chromosomes. Horizontal lines indicate two types of significance thresholds. The 'pooling noise' significance threshold was based on a simulation of the bulking process (see Figure 2). The 'platform noise' significance threshold was derived from an analysis of the distribution of values in the self comparison (bottom panel) and Bonferroni**-**adjusted for multiple comparisons (see section entitled 'Polymorphic marker identification' in *Materials and Methods*).

A plot of the allele-frequency difference between *mPub *bulks (40 plants each) vs. the chromosomal positions of the markers on a DArT consensus map [20] immediately confirmed that *mPub *was located on the long arm of chromosome 3H (Figure 3, top panel) [21]. The marker with the maximum allele-frequency difference (93.5%) was bPb-8978. A Steptoe/Morex map for chromosome 3H, built from markers overlapping between this and a previous study [20], confirmed that bPb-8978 was the closest marker to *mPub *at 4.6 cM distance. The Loess curve also peaked at this marker. These results underscore the mapping accuracy of DArT-BSA.

The *mPub *linkage signal was highly significant, peaking well beyond both the 'pooling noise' and the 'platform noise' threshold (Figure 3, top panel). A comparison of two randomly assembled bulks of 40 plants (Figure 3, middle panel) showed that bulks were not large enough to decrease the random fluctuations in allele frequencies to a level comparable to the apparent fluctuations caused by the baseline noise of the DArT platform (Figure 3 bottom panel). The SD of the allele-frequency difference was 12.0% in the random-bulk comparison, 12.5% in the *mPub*-bulk comparison (all chromosomes except 3H), but only 5.9% in the self comparison.

We conclude from this data that in the case of barley, it would be beneficial to bulk up to 150 DH plants for DArT-BSA. At this bulk size the extent of random fluctuations in allele frequencies introduced in the pooling process (Figure 2) become comparable to the approximate 'platform noise' of DArT-BSA (24% at *p *< 0.05 in the test experiment, but only 17% in the subsequent validation experiment).

#### Linkage decay in the vicinity of the target locus

**Figure 4:**
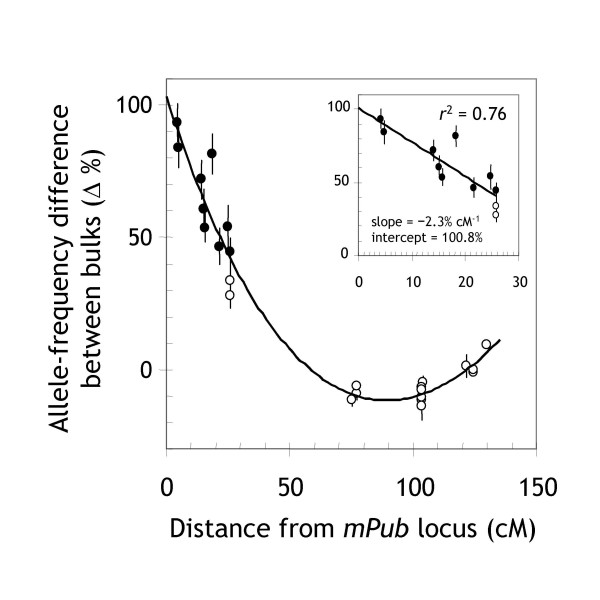
**Decay of linkage as a function of the genetic distance between markers and a trait locus**. The measured allele-frequency difference between two bulks (40 plants each) was plotted against the cM distance between chromosome-3H markers and the *mPub *locus. The inset displays a linear regression of the allele-frequency difference for markers within ± 30 cM from *mPub *on the cM distance from *mPub*. Black data points are significantly linked to *mPub *at the *p *< 0.05 level for 'pooling noise' (see Figure 2).

The 'linkage signal' decayed as the distance between markers and the target locus increased (Figure 4). A linear-regression analysis using markers within ± 30 cM of *mPub *indicated a 2.3% decrease per cM distance from the target locus (see inset in Figure 4). It is therefore possible to estimate the approximate cM distance of DArT markers from the target locus based on the following formula: cM distance ≈ 0.43 × % allele-frequency difference – 43. This relationship could be useful for analyzing populations derived from genetically close parents. In such situations, lower marker densities are expected and the approximate position of the target locus could be estimated from moderately linked markers based on this relationship.

Gel-based markers (RAPD, AFLP and SSR) compromise the efficiency and precision of BSA by generating discrete allelic data in a somewhat arbitrary and hence error-prone process (i.e., alleles are called 'present' or 'absent'). The latter is problematic in situations where the bulks contain different proportion of both parental alleles, for example in case of less than perfectly linked makers or for QTL with moderate effects. The ability of DArT-BSA to quantify the degree of linkage between markers and a target locus is a clear advantage in such cases.

#### Marker density

The *mPub *genome scan comprised 433 polymorphic markers (Table 1), that is, approximately one marker every 2.7 cM. This resolution is lower than the resolution afforded by the *Arabidopsis *SFP array [9,23]. However, SFP hybridization data tend to be quite variable, which makes a high marker density an important prerequisite for calculating robust allele-frequency difference estimates for genomic regions. More importantly, the precision with which a target locus can be mapped by BSA not only depends on the marker density but also on the number of crossover in the vicinity of the target locus. In experimental populations that are the result of a limited number of meioses the 'linkage peaks' will simply be too broad to make effective use of more than approximately 500 to 1,000 markers.

### Validation experiment

Having established that DArT-BSA accurately identifies the known genomic location of a model trait, we continued to validate the method by attempting to map an Al-tolerance locus in a Dayton/Zhepi2 DH population (Wang et al., submitted). For this purpose we 'relaxed' the conditions of analysis in two ways to accommodate more typical experimental designs. First, we pooled genomic-DNA samples (for a comparison against pooled genomic representations). Second, we only pooled 20 plants per bulk, not an uncommon bulk size in this sort of experiments.

#### Genomic-DNA samples can be bulked directly

Because DArT-BSA compares the abundance of alternative alleles between bulks in a quantitative manner (Figure 1), it is important to make sure that individual plants contribute equally to bulks, particularly when working with small bulk sizes. Presumably, the most robust way to achieve this is to bulk representations derived from individual samples of genomic DNA. Direct bulking of genomic-DNA samples, however, produces virtually identical estimates of allele-frequency differences (*r *= 0.91; Additional File 2). It may well be tolerable to bulk equal amounts of plant material before DNA extraction, although we did not test this method experimentally.

#### DArT-BSA identifies an Al-tolerance locus on chromosome 4H

**Figure 5:**
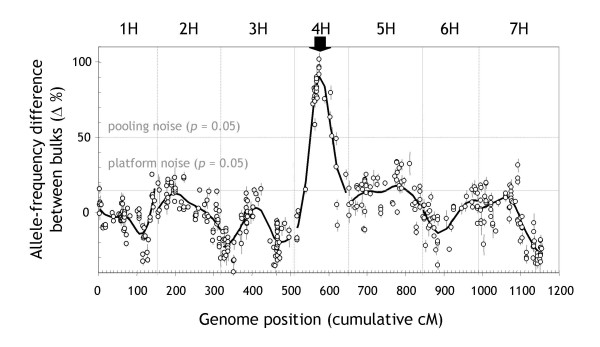
**DArT-BSA genome scan for Al tolerance in the Dayton/Zhepi2 DH population**. The difference in the frequency of the Dayton allele between two bulks with contrasting Al tolerance (20 plants each) is shown as a function of the cM positions of markers previously incorporated into a DArT consensus map for barley (446 of 490 polymorphic markers) [20]. The vertical lines within the panel denote borders between individual chromosomes. Horizontal lines indicate the 'pooling noise' and 'platform noise' significance thresholds as defined in Figure 3, using Dayton and Zhepi2 instead of Steptoe and Morex. The black arrow indicates the approximate position of the *Alt *locus which was identified in other populations as the principal locus conferring Al resistance in barley [24–30].

The DArT-BSA scan for Al tolerance in the Dayton/Zhepi2 population revealed a highly significant peak on chromosome 4H, despite the elevated 'pooling-noise' threshold (50%; *p *< 0.05) due to the small bulk sizes used (Figure 5). The position of the peak is consistent with the location of an Al-tolerance locus (*Alt*) on 4HL, which appears to mediate Al-activated citrate secretion from roots, and has already been identified in several barley populations [24-30].

The *Alt *locus was previously mapped between SSR markers Bmag490 on the proximal side and HVM68 on the distal side [29,30]. These two SSR markers have also been incorporated into a DArT linkage map of the Dayton/Zhepi2 population where they span a small 0.8-cM region (see Additional File 2 in [20]). The marker that showed the greatest allele-frequency difference in the DArT-BSA scan (101.7%; bPb-6872) co-segregated with Bmag490, which implies that it must have been closer than 0.8 cM from the *Alt *locus. The Loess curve peaked another 4.2 cM proximal of the Bmag490/bPb-6872 locus (Figure 5).

These results are consistent with the performance of DArT-BSA in the Steptoe/Morex model experiment conducted under technically more stringent experimental conditions (Figure 3). In both experiments the position of the target locus was mapped with at least 5 cM precision. A third experiment not reported here identified markers linked to a disease-resistance locus that was subsequently confirmed by conventional linkage analysis.

## Conclusion

Given that DArT arrays are already available for two dozens of plant and fungal species [4,13-18] and can be rapidly developed for new species of any ploidy level with limited resources [19], we extrapolate from the results of this study that DArT should prove useful as a generic platform for quantitative BSA in plants. DArT markers in established arrays for a number of important crops are being sequenced, thus providing instant access to sequence anchor(s) for any DArT-BSA-tagged character when sufficient genomic resources become available in the future.

## Methods

### Populations and targeted traits

Two DH populations derived from the F_1 _of bi-parental crosses between barley cultivars were used for this study. The Steptoe/Morex population segregated for the morphological marker 'pubescent leaf blades' (*mPub*) [21]. The Dayton/Zhepi2 population segregated for Al tolerance (Wang et al., submitted). DNA was prepared from individual plants using a CTAB method [31,32].

### Bulks

Individual DH plants derived from the Steptoe/Morex cross were classified as 'pubescent' and 'hairless' according to their *mPub *allele [33] and assigned to two pairs of contrasting bulks (20 or 40 plants each). The DH progeny from the Dayton/Zhepi2 cross was assessed for Al tolerance using a nutrient solution-culture method [26]. The roots were exposed to 15 μM of Al for 3 days and stained with 0.1% (w/v) eriochrome cyanine R. The seedlings were then visually scored as 'sensitive' or 'tolerant' as described previously [29] and assigned to one of two contrasting bulks of 20 plants each.

'Representation bulks' were prepared by mixing the genomic representations prepared from individual genomic DNA samples (see section entitled *DArT assays *below). 'Genomic bulks' were prepared by mixing the genomic DNA samples (~1 μg each) themselves (Dayton/Zhepi2 population only).

### Experimental design

A BSA experiment typically comprised four comparisons between individual DNA samples or DNA pools. First, phenotypically contrasting bulks (either of the 'genomic' or the 'representation' type) were compared by simultaneously assaying them on the same DArT array. Second, representations prepared from the two parents were compared on a separate array. Third, a pair of 'representation bulks' derived from two randomly assorted DNA pools ('random bulks') were assayed on another array to evaluate the impact of stochastic effects associated with bulking. Fourth, two identical aliquots of a 1:1 mixture of the two parents ('parent mixture') were compared against each other to quantify the platform (technical) noise of the DArT assays. Each pair of samples ('genomic bulks', 'representation bulks', 'parents', 'random bulks', 'parent mixture') was independently analyzed up to eight times on separate arrays. Half of these replicate assays were performed by swapping the cy3 and cy5 fluorescent dyes used to label the representation pairs compared against each other (see next section).

### DArT assays

*Pst*I/*Bst*NI representations of genomic DNA samples (from individual plants or 'genomic bulks') were prepared as described previously [4]. The representations produced from individual DNA samples were either bulked according to *mPub *or Al tolerance ('representation bulks') or pooled randomly ('random bulks'). All representations and 'representation bulks' were purified, labeled with cy3 or cy5 and hybridized to DArT arrays together with the FAM-label polylinker of the vector that had been used to clone the fragments printed on the arrays [3,14]. The DArT arrays contained 48 sub-arrays with 2,304 polymorphism-enriched clones printed in duplicate and 384 control clones, each printed six times (6,912 array features in total) [20]. The arrays had been printed with a MicroGrid II arrayer (Biorobotics, Cambridge, UK) on SuperChip poly-L-lysine slides (Erie Microarray, Portsmouth NH, USA) using DArT-spotter, a thoroughly optimized buffer for heavy-duty microarray printing (Wenzl et al. in preparation). After an overnight hybridization at 62°C, the arrays were washed and scanned with 10-μm resolution at 543 nm (cy3), 633 nm (cy5) and 488 nM (FAM) on a LS300 confocal laser scanner (Tecan, Grödig, Austria) [3,14].

### Array-data analysis

Array images were analyzed with DArTsoft 7.4 (Diversity Arrays Technology P/L, Canberra, Australia). The program automatically recognized array features using a seeded-region-growth algorithm and reported, for each fluorescent channel, the average and SD of pixel intensities within and around each array feature, the fraction of saturated pixels within each feature and the number of pixels of each feature, amongst other parameters (Cayla et al. in preparation). The logarithm of the ratio between the two background-subtracted averages of feature pixels in the cy3 and the cy5 channel (log_2 _[cy3/cy5]) was used as a measure of the difference in abundance of the corresponding DNA fragment in the two representations hybridized to an array. The log_2 _[cy3/FAM] and log_2 _[cy5/FAM] values, approximate measures of the amount of hybridization signal per amount of DNA spotted on the array, were used for quality-control purposes (see next section).

#### Whole array quality-filtering

Arrays were rejected if the average correlation of either the log_2 _[cy3/FAM] or log_2 _[cy5/FAM] values of non-polymorphic clones (as identified below) with the corresponding values from all other arrays in an experiment was smaller than 0.9. One out of 68 arrays (1.5%) was removed this way.

#### Array feature quality-filtering

Some array features hybridized weakly in both fluorescent channels, either because of an insufficient amount of DNA printed on the array or because the corresponding DNA fragments were not captured in the genomic representation of either parent. Therefore, features with signal-to-noise ratios (the background-subtracted average of feature pixels divided by the SD of local-background pixels) below 5 in both fluorescent channels were removed from further analysis. The remaining features were accepted if the coefficient of variation of their pixel intensities was smaller than 70% in at least one of the two channels, if less then 20% of feature pixels were saturated in both channels, if the SD of background pixels was smaller than 5 times the array median in both channels, and if their size (pixel number) was at least 30% of the array median. Overall, this quality-filtering procedure removed 12.3 ± 0.7% of all array features (mean ± SD across all experiments).

#### Hybridization-intensity normalization and averaging

The SD of the central 90% of all log_2 _[cy3/cy5] feature values on an array was scaled to the average SD of the group of arrays hybridized to the same type of DNA samples or pools. The average of the central 90% of all features on each array was adjusted to zero. Subsequently, the normalized/scaled log_2 _[cy3/cy5] values of replicate features (both within and across arrays hybridized with identical sample pairs) were averaged to obtain clone values (values derived from dye-swap arrays were multiplied by -1). For clones with at least 6 remaining replicate features, only the central 66% of values were averaged. Clones with less than 25% of replicate features left and the 3% of clones with the highest across-replicate-feature SD of log_2 _[cy3/cy5] were removed from further analysis. In this manner the best 92.9 ± 0.1% of all clones on the array were selected as potential markers for BSA analysis (mean ± SD across all experiments).

#### Polymorphic marker identification

Polymorphic clones (markers) were selected from the set of quality-filtered clones, using a two-tiered approach. In step one, a normal distribution-based significance threshold for log_2 _[cy3/cy5] clone values was established (typically at *p *< 0.0001) to detect outliers in the comparison between two aliquots of the parent mixture. Polymorphic markers were identified in the comparison between the parents based on this log_2 _[cy3/cy5] threshold, after it was adjusted by the ratio of the mean between-replicate-feature SD in the two types of comparisons. Potentially unstable polymorphic clones were identified by searching for polymorphic clones that were present in the tails of the log_2 _[cy3/cy5] distribution in the comparison between two aliquots of the parent mixture (*p *< 0.05). They were excluded from further analysis.

For step two, the log_2 _[cy3/cy5] values obtained in the 'representation/genomic bulks', 'random bulks' and 'parent mixture' comparisons were referenced against (divided by) the corresponding values measured in the comparison between parents. Ratios between two groups of log_2 _[cy3/cy5] values (derived from replicated array features) were computed using weighted jackknifing [34]. The resulting values for relative hybridization contrast (i.e., the hybridization contrast as a percentage of the contrast between the parents) were accepted if their standard error was smaller than 10%. The values obtained in the comparison of two aliquots of the parents mixture were screened for outliers using a normal distribution-based significance threshold of *p *= 0.05. Outlier markers were excluded.

### Allele-frequency determination and simulation

A subset of the markers that were identified as polymorphic in the Steptoe vs. Morex comparisons had previously been incorporated into a linkage map for this population [20]. For each of these markers, the difference in the frequency of the Steptoe allele between the contrasting *mPub *bulks of 40 plants was calculated from the segregation data after inferring missing data and removing likely genotyping errors (see section entitled *Distance of DArT markers from mPub *below).

The probability of detecting spurious linkages when analyzing bulks of limited size was estimated by evaluating the distribution of the maximum difference in allele frequency in 10,000 comparisons between random bulks consisting of varying numbers of simulated barley DH genotypes (10, 20, 40 and 80). The latter were generated by randomly seeding seven chromosome telomeres with one of the two parental alleles, followed by propagating the seeded genotypes along 59 additional chromosomal loci based on a Markov chain with a constant 3% transition probability (i.e., assuming 60 equidistant loci on 7 chromosomes of approximately 180 cM length each).

### Marker positions on linkage maps

#### Distance of DArT markers from mPub

Segregation data of DArT markers previously mapped to chromosome 3H in the Steptoe/Morex population were combined with the segregation pattern of *mPub *[20,33]. The 3H map was then re-optimized using the RECORD algorithm [35] and missing data were inferred from neighboring markers. Potential genotyping errors were identified as described previously [36] (LOD_error _> 4) and replaced with missing data (< 0.2% of allele calls). Map distances between *mPub *and all other markers on chromosome 3H were then computed by adding Kosambi cM distances between adjacent markers.

#### Genome-scan display

The relative contrast in hybridization intensity of a subset of markers that had previously been incorporated into a DArT consensus map for barley [20] were plotted against the markers' positions in the barley genome. A Loess curve was fitted to each of the chromosomes to visualize changes in allele frequency across chromosomes.

## List of abbreviations

AFLP, amplified fragment length polymorphism; Al, aluminum; *Alt*, aluminum-tolerance locus; BC, backcross; BSA, bulked segregant analysis; CTAB, cetyl trimetyl ammonium bromide; DArT, diversity arrays technology; DH, doubled haploid; FAM, 5,6-carboxy-fluorescein; LOD_error_, logarithm of odds value for genotyping error; Loess, locally-weighted scatterplot smoothing; *mPub*, pubescent leaf blades locus; QTL, quantitative trait locus/loci; RAPD, random amplified polymorphic DNA; RIL, recombinant inbred line(s); SD, standard deviation; SFP, single feature polymorphism; SNP, single nucleotide polymorphism; SSR, simple sequence repeat

## Authors' contributions

PW designed and performed the DArT-BSA assays, analyzed the data and drafted the manuscript. HR performed some Al-tolerance bioassays and supervised molecular and physiological work on the Dayton/Zhepi2 population. JW isolated DNA of the Dayton/Zhepi2 population, performed the Al-tolerance bioassays and assembled the Al-tolerant and sensitive bulks. MZ provided the Dayton/Zhepi2 DH population. EH provided ongoing intellectual input to the design and development of the barley DArT array and edited the manuscript. AK provided overall guidance during the development of DArT for barley, including its deployment in BSA and co-designed and edited the manuscript. All authors read and approved the final manuscript.

## Supplementary Material

Additional File 1 **Influence of the hybridization contrast between parental alleles on the precision of estimating allele-frequency equality**. PDF file with a chart displaying the relationship between the apparent allele-frequency differences, measured by comparing two identical aliquots of a 1:1 mixture of Steptoe and Morex, and the hybridization contrasts between alternative alleles. All markers present in a Steptoe/Morex DArT map [20] were included in this figure. The SD of groups of markers in allelic-contrast bins of 0.5 units on the log_2 _[cy3/cy5] scale are included. Click here for file

Additional File 2 **Comparison between two bulking strategies**. PDF file with a chart displaying, for each of 490 markers, the difference of the Dayton allele frequency between Al-tolerant and Al-sensitive bulks of 20 Dayton/Zhepi2 DH plants (Δ %), measured using two alternative methods: (1) by pooling representations prepared from individual DNA samples (horizontal axis), and (2) by preparing two representations from genomic-DNA pools. The dotted line denotes equality between the two alternative methods. Click here for file
