# Supplementary material for: A DArT platform for quantitative bulked segregant analysis
Source: BMC Genomics. 2007 Jun 28;8:196. doi: 10.1186/1471-2164-8-196 (PMC1920522; doi:10.1186/1471-2164-8-196)

Additional file 1

Influence of the hybridization contrast between parental alleles on the precision of estimating allele-frequency equality

The chart displays the relationship between the apparent allele-frequency differences, measured by comparing two identical aliquots of a 1:1 mixture of Steptoe and Morex, and the hybridization contrasts between alternative alleles. All markers present in a Steptoe/Morex DArT map [25] were included in this figure. The SD of groups of markers in allelic-contrast bins of 0.5 units on the $\log_2$[cy3/cy5] scale are included.

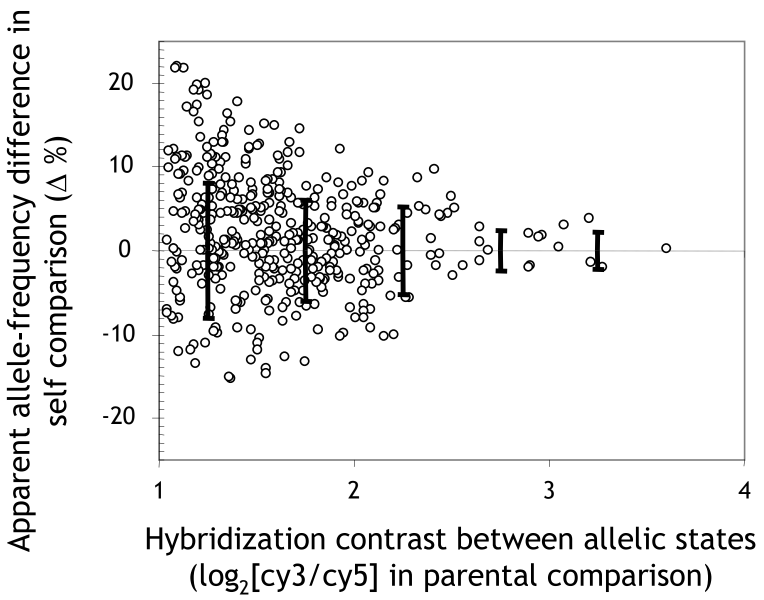

Supplement: Additional File 1 — Influence of the hybridization contrast between parental alleles on the precision of estimating allele-frequency equality. PDF file with a chart displaying the relationship between the apparent allele-frequency differences, measured by comparing two identical aliquots of a 1:1 mixture of Steptoe and Morex, and the hybridization contrasts between alternative alleles. All markers present in a Steptoe/Morex DArT map [20] were included in this figure. The SD of groups of markers in allelic-contrast bins of 0.5 units on the log2 [cy3/cy5] scale are included. [file 1471-2164-8-196-S1.pdf]
