# Supplementary material for: A DArT platform for quantitative bulked segregant analysis
Source: BMC Genomics. 2007 Jun 28;8:196. doi: 10.1186/1471-2164-8-196 (PMC1920522; doi:10.1186/1471-2164-8-196)

Additional file 2

Comparison between two bulking strategies

The chart displays, for each of 490 markers, the difference of the Dayton allele frequency between Al-tolerant and Al-sensitive bulks of 20 Dayton/Zhepi2 DH plants (□ %), measured using two alternative methods: (1) by pooling representations prepared from individual DNA samples (horizontal axis), and (2) by preparing two representations from genomic-DNA pools. The dotted line denotes equality between the two alternative methods.

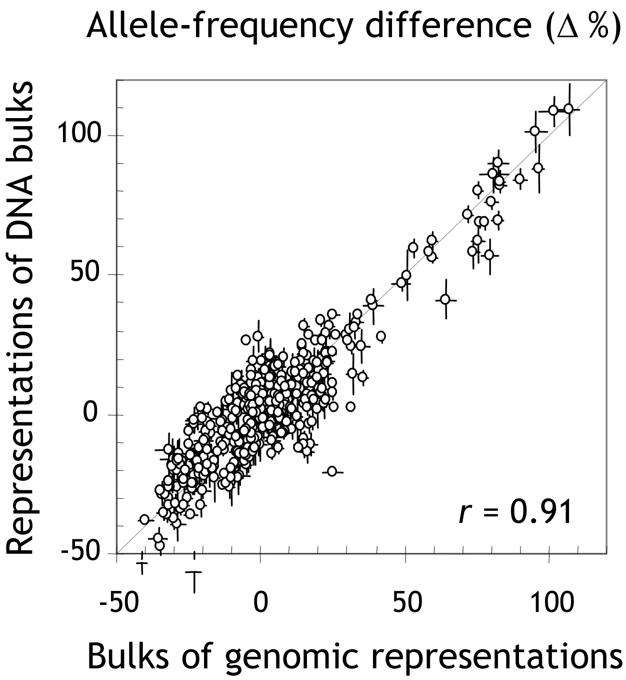

Supplement: Additional File 2 — Comparison between two bulking strategies. PDF file with a chart displaying, for each of 490 markers, the difference of the Dayton allele frequency between Al-tolerant and Al-sensitive bulks of 20 Dayton/Zhepi2 DH plants (Δ %), measured using two alternative methods: (1) by pooling representations prepared from individual DNA samples (horizontal axis), and (2) by preparing two representations from genomic-DNA pools. The dotted line denotes equality between the two alternative methods. [file 1471-2164-8-196-S2.pdf]
